# 

**Published:** 1896-07

**Authors:** 


### INDEX—VOL. XII., 1896-'97.

A Case of Foreign Bodies in the Stomach, with Treatment . . . . 666
Asexualization for Crime . . . . . . . . . . . . . . . . . . . . . . . 684
Atlanta Freak, Th . . . . . . . . . . . . . . . . . . . . . 266
American Public Health Association . . . . . . . . . . 117, 133
Abortion of Smallpox . . . . . . . . . . . . . . . . . . . . . 83
Another Clincher . . . . . . . . . . . . . . . . . . . . . . 38
Association Bill, T e . . . . . . . . . . . . . . . . . . . . . 514
Austin District Medical Society . . . . . . . . . . . . . . . . 151
An Unmerited Slu . . . . . . . . . . . . . . . . . . . . . 144
An Embryo State Training School . . . . . . . . . . . . . . 259
Associated Physicians and Surgeons of Santa Clara Valley . . . . 447
An Unfortunate Young Man . . . . . . . . . . . . . . . . . . 457

Brazos Valley Medical Association . . . . . . . . . . . . . . 612
Best are Barred, The . . . . . . . . . . . . . . . . . . . . . 335
Bubonic Plague; Malignant Polyadenitis . . . . . . . . . . . . 618
Book Notices, 582-586, 522-524, 466-471, 402-405, 343, 212-217, 156, 52, 694-696
Broncho-Pneumonia . . . . . . . . . . . . . . . . . . . . . 57
Brazos Valley Medical Association . . . . . . . . . . . 183, 322, 678

Case of Mostoiditis Complicating Purulent Otitis Media Cured by Enlarging the Drum; Perforation and Syringing the Tympanitic Cavity . . . . . . . . . . . . . . . . . . . . . 653
Central Texas Medical Association . . . . . . . . . . . . . . . 374
Case of Chondro-Sarcoma of the Chest, A . . . . . . . . . . . 415
Clinical Lecture . . . . . . . . . . . . . . . . . . . . . . . 422
Century of Vaccination . . . . . . . . . . . . . . . . . 246, 258
Castration . . . . . . . . . . . . . . . . . . . . . . . . . 440
Civilization and Tuberculosis . . . . . . . . . . . . . . . . . 314
Case of Protracted Labor, A . . . . . . . . . . . . . . . . . 231
Conservatism in Gunshot Wounds of the Abdomen . . . . . . . 235
Cumulative Action of Alcohol . . . . . . . . . . . . . . . . . 268
Cheap Learning. . . . . . . . . . . . . . . . . . . . . . . . 688
Chronic Interstitial Nephritis . . . . . . . . . . . . . . . . . 161
Chronic Constipation . . . . . . . . . . . . . . . . . . . . . 166
Cure of Deviations of the Nasal Septum . . . . . . . . . . . . 173
Child Crying in Utero . . . . . . . . . . . . . . . . . . . . . 111
Chemismus of the Stomach . . . . . . . . . . . . . . . . . . 538
Consumption in San Antonio . . . . . . . . . . . . . . . . . . 475
Chronic Diffuse Nephritis Without Exudation . . . . . . . . . . 479

Department of Public Health in the U. S. Government, A . . . 505
Death of Dr. Jerome Cochran . . . . . . . . . . . . . . . . . 143
Dr. Williamson Henderson Wilkes . . . . . . . . . . . . . . . 148

Dr. J. B. Hamilton and "One Walter Wyman" . . . . . . . . . . 635
Dr. J. B. Hamilton . . . . . . . . . . . . . . . . . . . . . . . . 261
Don't Monkey With the Buz-Saw . . . . . . . . . . . . . . . . . 267
Degenerate Son, A . . . . . . . . . . . . . . . . . . . . . . . . 327
Dr. Paschal's Paper . . . . . . . . . . . . . . . . . . . . . . . 331
Dr. Hamilton Again . . . . . . . . . . . . . . . . . . . . . . . 334
Doctor's Wife, The . . . . . . . . . . . . . . . . . . . . . . . 506
Dr. Osborn's Carbuncle Treatment . . . . . . . . . . . . . . . . 364

Effect and Result of Sero-Therapy in Tuberculosis . . . . . . . 8
Expediency vs. Principle . . . . . . . . . . . . . . . . . . . . 25
Ethics of a Mixed Board of Medical Examiners; Letter from Prof. N. S. Davis, M. D. . . . . . . . . . . . . . . . . . . . . . . . 29
Euthanasia . . . . . . . . . . . . . . . . . . . . . . . . . . . 565
East Texas Medical Society. Quarterly Meeting . . . . . . . . . 82

For the Benefit of the Unfortunate Young Man. Drs. F. B. McRae, J. W. Cole, J. S. W., Benj. Frenkel and W. H. Lancaster . . . . . . . . . . . . . . . . . . . . . . . . . . 488, 496
Fiftieth Anniversary of the American Medical Association . . . 610
From Committee on Medical Societies . . . . . . . . . . . . 117, 133
Florid Mendacity. Why Physicians Do Not Advertise . . . . . 615
Farewell Address to the Class of 1896, Marion Sims Medical College. Prof. A. C. Bernays . . . . . . . . . . . . . . . . . 43
French Revolutionist, Jean Paul Marat, The . . . . . . . . . . 598
Family Jars . . . . . . . . . . . . . . . . . . . . . . . . . . 644

Gunshot Fracture of Clavicle. Notes on Case . . . . . . . . . . 443
Gems of Journal Literature . . . . . . . . . . . . . . . . . . . 88

Hypnotism as She is Taught in Chicago . . . . . . . . . . . . . 263
Help Wanted . . . . . . . . . . . . . . . . . . . . . . . . . . 445
Hereditary Insanity . . . . . . . . . . . . . . . . . . . . . . 300
Hypertrophy of the Pharyngeal Tonsil . . . . . . . . . . . . . 18

Imperforate Hymen . . . . . . . . . . . . . . . . . . . . . . . 239
Is Malaria a Land or Water Disease . . . . . . . . . . . . . . . 687
Is There a Typho-Malarial Fever? . . . . . . . . . . . . . . . 246
International and Great Northern . . . . . . . . . . . . . . . 271

Journal's Exchange Bureau . . . . . . . . . . . . . . . 155, 280, 211
Journal's Own Page . . . . . . . . . . . . . . . . 102, 280, 211, 452

Letter from Dr. Q. C. Smith, including letter of Dr. McLean . . 113
Lunatic Asylums and the Medical Colleges . . . . . . . . . . . 238
Land of Montezuma . . . . . . . . . . . . . . . . . . . . . . . 272
Let Him Who is Without Sin Cast the First Stone . . . . . . . 41
Logical Sequence, A . . . . . . . . . . . . . . . . . . . . . . 641
Legislation Run Mad . . . . . . . . . . . . . . . . . . . . . . 643
Legislation. The Undertaker's Bill and Other Bills . . . . . . 575
Local Treatment of Diphtheria, and Other Items . . . . . . 246, 258

Medical Education in Texas . . . . . . . . . . . . . . . . . . . . . . . 664
Medical News and Miscellany . . 645-651, 579, 47, 340-342, 463-466, 398-402, 204-211, 274-280, 99, 151, 517-522, 691-694.
Meharry Medical College . . . . . . . . . . . . . . . . . . . . . . . 514
Medico-Legal Society of Chicago. Dr. Lydston's Paper . . . . . 117
Meeting of American Association of Obstetricians and Gynecologists . . . . . . . . . . . . . . . . . . . . . . . . . . . . 117, 133
Mississippi Valley Medical Association . . . . . . . . . . . . . 117, 133
Medlcal Expert Testimony . . . . . . . . . . . . . . . . . . . . . . 351
"Mirror" Reflects, The . . . . . . . . . . . . . . . . . . . . . . . 384
Modern Treatment of Acute Inflammation of the Middle Ear . . 175
Medical Law, The, A Suggestion for Relief . . . . . . . . . . . 197
Medical Sensation, A . . . . . . . . . . . . . . . . . . . . . . . . 269
Modern Treatment of Diphtheria in Private Practice . . . . . . 324
Medical Legislation . . . . . . . . . . . . . . . . . . . . . . . . . 339
Meeting of the Missouri State Medical Association . . . . . . . 557

New York Academy of Medicine. Section in Orthopædic Surgery 607, 676
New Treatment for Consumption, A . . . . . . . . . . . . . . . . 571
New Method of Treating Carbuncle . . . . . . . . . . . . . 302, 335
Normal Labor . . . . . . . . . . . . . . . . . . . . . . . . . . . . 441
Mew Treatment of Carbuncle. Reply to Dr. Morris . . . . . . . 445
No Row . . . . . . . . . . . . . . . . . . . . . . . . . . . . . . . 456
Northwest Medical Associations . . . . . . . . . . . . . . . . . 670
Nasal Catarrh . . . . . . . . . . . . . . . . . . . . . . . . . . . . 459
New Stone Searcher, A . . . . . . . . . . . . . . . . . . . . . . . 69
New Cure for Gonorrhea . . . . . . . . . . . . . . . . . . . . . . 77
Nude in Medical Journalism, The . . . . . . . . . . . . . . . . . 144
Notes on Some of the Newer Remedies Used in Skin Diseases . . 503

Our Duty as Physicians . . . . . . . . . . . . . . . . . . . . . . 360
Osteo-Myelitis of the Tibia and Fibula . . . . . . . . . . . . . 169
On the Use of Electrolysis in Stenosis of the Cervix Uteri . . . 540
On the Proper Treatment of Consumption . . . . . . . . . . . . 1
Our 12th Voyage . . . . . . . . . . . . . . . . . . . . . . . . . . 45

Publisher's Notes. New Advertisements of Medical and other Colleges . . . . . . . . . . . . . . . . . . . . . . . . . . . . . 53
Publisher's Notes . . . . 343-350, 586-592, 282-288, 651-652, 471-474, 103 217-224, 405-514, 159, 524-532, 696.
Puerperal Eclampsia; Its Etiology and Treatment . . . . . . . . 500
Penny Wisdom; Mistaken Philanthropy . . . . . . . . . . . . . . 511
Puerperal Eclampsia . . . . . . . . . . . . . . . . . . . . . . . . 109
Public Hygiene—Resolutions and Suggestions . . . . . . . . . . . 667
Protest, A . . . . . . . . . . . . . . . . . . . . . . . . . . . . . 116
Pan-American Medical Congress . . . . . . . . . . . . . . . . . . 78
Practice Much Affected, A . . . . . . . . . . . . . . . . . . . . . 146
Proper Treatment of Abortion, The. Reply to Dr. Smith's Criticism . . . . . . . . . . . . . . . . . . . . . . . . . . . . . . . 550
Pretext, The, Expediency . . . . . . . . . . . . . . . . . . . 35, 36

Quarantine Question, The . . . . . . . . . . . . . . . . . . . . 638
Quackery Rampant . . . . . . . . . . . . . . . . . . . . . . 94

Report of a Case of Skin Grafting . . . . . . . . . . . . . . . . 594
Recognition of Illegal Practice, The . . . . . . . . . . . . . . 242
Rheumatism . . . . . . . . . . . . . . . . . . . . . . . . . 434
Richmond Academy of Medicine and Surgery . . . . . . . . . . . 316
Recent Meeting of the American Ophthalmological Society . 178, 181
Relation of Examining Boards to State, etc. . . . . . . . . . . . 76
Richmond Academy of Medicine and Surgery . . . . . . . . . . 366
Rational Medicine; Assisting Nature . . . . . . . . . . . . . . 393

Saunders, Prof. Bacon, M., D., President of the Texas State Medical Association . . . . . . . . . . . . . . . . . . . . . . . 669
Spider Bite . . . . . . . . . . . . . . . . . . . . . . . . . 493
Suicide . . . . . . . . . . . . . . . . . . . . . . . . . . . 507
State Hospital, A. . . . . . . . . . . . . . . . . . . . . . . 512
Strangulated Hernia . . . . . . . . . . . . . . . . . . . . . 135
Situation, The . . . . . . . . . . . . . . . . . . . . . . . 142
Screw Tightens, The . . . . . . . . . . . . . . . . . . . . . 147
Suicide Considered a Mental Epidemic . . . . . . . . . . . . . . 376
State Health Officer's Report . . . . . . . . . . . . . . . . . 396
Serum Therapy Antedated. Letter from Dr. J. W. Daniel . . . 178
Serum Therapy in Tuberculosis . . . . . . . . . . . . . . . . . 186
Smythe, Dr., Letter from . . . . . . . . . . . . . . . . . . . 392
Some of the Unsuspected Effects of the Bromides . . . . . . . . 189
Some More Quackery and Bogus Diplomas . . . . . . . . . . . . 203
Something on Medical Mistakes . . . . . . . . . . . . . . . . . 289
South Texas Medical Society . . . . . . . . . . . . . . . . . . 323
Strike Now for a Medical Bill . . . . . . . . . . . . . . . . . 453
State Care of Idiots . . . . . . . . . . . . . . . . . . . . . 547
Supplied Blood in Extremis. A Killer of Septicæmia and Syphilitic Virus . . . . . . . . . . . . . . . . . . . . . . . . . 563
Southern Surgical and Gynecological Association . . . . . . . . 243
Soft Thing, A . . . . . . . . . . . . . . . . . . . . . . . . 267
Song of the Peripatetic, The . . . . . . . . . . . . . . . . . . 269
Suprapubic Lithotomy . . . . . . . . . . . . . . . . . . . . . 599
South Texas Medical Association . . . . . . . . . . . 609, 611, 682
Southeast Texas Medical Association . . . . . . . . . . . . . . 614
Some Further Perversions of Facts . . . . . . . . . . . . . . . 90

Tyler Medical Society . . . . . . . . . . . . . . . . . . 117, 133
Texas Health College Evil Again . . . . . . . . . . . . . . . . 496
"Texas Health College" Matter, The . . . . . . . . . . . . . . 577
Texas Health College Diploma . . . . . . . . . . . . . . . . . 116
Tri-State Medical Society of Alabama, Georgia and Tennessee. 183, 186
Transportation Arrangements for the Pan-American Medical Congress . . . . . . . . . . . . . . . . . . . . . . . . . 183, 186
Tri-State Medical Society . . . . . . . . . . . . . . . . . 117, 133
Treatment of Abortion . . . . . . . . . . . . . . . . . . . . . 62

"Told You So" . . . . . . . . . . . . . . . . . . . . . . . . . 87
Texas Medical News, The: Explanation, etc. . . . . . . . . . . . . 91
Triune Man, The . . . . . . . . . . . . . . . . . . . . . . . . 603
Texas State Medical Association, The . . . . . . . . . . . . . . 629
That Foolish Legislature . . . . . . . . . . . . . . . . . . . . 633
Too Good to Keep. Differential Diagnosis . . . . . . . . . . . 240
Transactions Texas State Medical Association . . . . . . . . . 274
Texas State Medical Association—Preliminary Announcement and Programme . . . . . . . . . . . . . . . . . . . . . . . . 558
Treatment of Abortion, The . . . . . . . . . . . . . . . . . . . 429
Treatment of Warty Growths on the Genitals, The . . . . . . . 451
Typho-Malarial Fever . . . . . . . . . . . . . . . . . . . . . . 309
Texas State Medlcal Association. Announcement of Officers of Sections for 1896-'97 . . . . . . . . . . . . . . . . . . . . . 12
Texas State Medical Association. A Card to Members of Local Societies . . . . . . . . . . . . . . . . . . . . . . . . . . 23
That Resolution . . . . . . . . . . . . . . . . . . . . . . . . . 36
Things Every Doctor Should Know . . . . . . . . . . . . . . . . 382
Treatment of the Throat, Nose and Ear in Scarlet Fever . . . . 388
Tape Worm Diagnosis in Urinalysis . . . . . . . . . . . . . . . 112
Texas State Medical Association, The . . . . . . . . . . . . . . 498

Use of Steam in Diseases of the Uterus . . . . . . . . . . . . 356
Uterine Hemorrhage Following Abortion . . . . . . . . . . . . 385
Umbilical Hemorrhage . . . . . . . . . . . . . . . . . . . . . . 70

Vaginal Ligation of the Uterine Arteries for Uterine Fibromata. 502
Veto of the Divorce Bill . . . . . . . . . . . . . . . . . . . . 689
Visceral Neurosis (Neuralgia) . . . . . . . . . . . . . . . . . 533
Value of Abdominal Belt After Coeliotomies . . . . . . . . . . 64
Vivisectionists Scared Up . . . . . . . . . . . . . . . . . . . . 81

Warm Discussion, A . . . . . . . . . . . . . . . . . . . . . . . 455
Water the Source of Malarial Poisen . . . . . . . . . . . . . . 655
Would Assexualization of Chronic Criminals, Sexual Perverts and Hereditary Defectives Benefit Society and Elevate the Human Race? . . . . . . . . . . . . . . . . . . . . . . . . . . . . 225
Worm Turns, The . . . . . . . . . . . . . . . . . . . . . . . . 84
Where is the Loop Hole . . . . . . . . . . . . . . . . . . . . . 200
What Sanitation Has Done for Life . . . . . . . . . . . . . 246, 258

